# HIV AIDS and substance abuse primary prevention in minority adolescents

**DOI:** 10.1186/1742-4690-9-S1-P128

**Published:** 2012-05-25

**Authors:** John Wodarski, Sam MacMaster

**Affiliations:** 1 University of Tennessee, Knoxville, USA

## Introduction

The research prevention project entitled “HIV/AIDS and Substance Abuse Primary Prevention in Minority Adolescents”, funded by the Substance Abuse and Mental Health Services Administration (SAMHSA), targets minority male and female adolescents 12-17 years old in the implementation and evaluation of a program designed to prevent adolescents from engaging in substance abuse and sexual activities which place them as risk for contracting the HIV/AIDS virus. The five-year intervention will serve 750 adolescents and 750 parents.

## Materials and methods

The program combines the effects of a program that consists of two evidence-based primary components: 1) refusal skills training and education for the adolescent focusing on issues relating to sexuality and substance abuse; and 2) a family prevention educational and skills component involving the parent(s) of the participating adolescents. The small group educational techniques employed with the adolescent component are based on the Teams-Games-Tournaments (TGT) Alcohol Prevention curriculum, cited as a Model Program in SAMHSA’s National Registry of Effective Programs and Practices (NREP) and as a Model Program by the Office of Juvenile Justice and Delinquency Prevention, and the Reducing the Risk (RTR) curriculum, both of which have been empirically evaluated as effective methods of teaching adolescent skills development in the areas of substance abuse and high-risk sexuality prevention. The parent prevention component is based on extensive research in which problem-solving skills and communication procedures have been used effectively with parents of adolescents.

## Results

The evaluation component of the project assesses participants (adolescents and parents) at baseline, post participation, and at 6-month follow-up periods. Dependent variables include the adolescents’ and parents’ knowledge of, attitudes toward, and behavior related to HIV AIDS and substance abuse. Additionally, adolescents’ self-efficacy and quality of peer and parental relationships are assessed. For all participating family members, conflict and communication measures are secured.

## Conclusion

Significant differences have been shown for adolescents and parents between baseline, post participation, and 6 month follow-up for all dependent variables. Training manuals provide program implementation requisites and materials to facilitate dissemination at local, state, and international levels.

